# An Alzheimer’s disease-associated mutant of *C. elegans* displays mechanosensory sensitivity following exposure to lavender extracts

**DOI:** 10.17912/micropub.biology.002220

**Published:** 2026-06-21

**Authors:** Melissa L. Schlein, Cayman A. Stephen, Guy A. Caldwell, Lukasz Ciesla, Kim A. Caldwell

**Affiliations:** 1 Department of Biological Sciences, University of Alabama, Tuscaloosa, AL, United States

## Abstract

*Lavandula angustifolia* (lavender) extract displayed antioxidant properties in mammalian studies and has been promoted as a candidate neurotherapeutic for Alzheimer's disease (AD). To better inform its clinical utility, we exposed wildtype ( N2 ) and *spr-4* mutant strains of *C. elegans* to extracts from this lavender species and examined animals for neurobehavioral changes in a mechanosensory phenotype. Importantly, *spr-4* encodes the worm ortholog of repressor element 1-silencing transcription factor (REST), an established genetic modifier of AD. While low concentrations of lavender did not alter behavioral responses, *spr-4* mutants selectively displayed neuronal vulnerability at the highest concentration tested, thereby revealing dose-responsive, lavender-associated neurotoxicity.

**Figure 1. Comparing the effect of lavender extracts on mechanosensory behavior:** Wildtype *C. elegans* N2 (blue bars) and *spr-4 ( by105 )* mutants (purple bars) were exposed to 0, 0.25, 1.0, and 2.6 mg/mL lavender extract. (A) anterior soft touch response and (B) posterior soft touch response. Error bars: s.e.m. N = 3; n = 30 per extract tested; Tukey's multiple comparison test (GraphPad Prism).

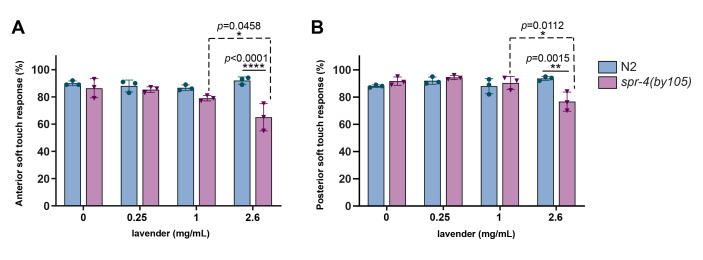



## Description

**Description**

Alzheimer's disease (AD) is associated with progressive memory loss, cognitive dysfunction, and neurodegeneration. It is also the most common cause of late-life dementia. Studies have shown that repressor element 1-silencing transcription factor (REST) is upregulated in healthy aging brains where it regulates a network of genes that resist cellular stress, cell death and AD pathology; in the brains of those affected with AD, REST is diminished (Calderone et al., 2003; Lu et al., 2014; McClelland et al., 2011). A *C. elegans* homolog of REST, *spr-4* , has been studied as a model for AD where the *spr-4 ( by105 )* nonsense mutation animals are more vulnerable to oxidative stress and *spr-4 ( by105 )* worms expressing SPR-4 or human REST are protected from oxidative stress (Lu et al., 2014). These data are consistent with the role of REST in stress resistance in the human body.

Natural products in the mint family, such as lavender, are often extracted as essential oils. Lavender extract has garnered interest as a neuroprotective agent against oxidative stress (Wang et al., 2012). &nbsp;The World Federation of Societies of Biological Psychiatry (WFSBP) and the Canadian Network for Mood and Anxiety Disorders (CANMAT)&nbsp;developed guidelines for use of nutraceuticals and phytochemicals in major psychiatric disorders (Sarris et al., 2022). Lavender was supported in these guidelines, to varying degrees, for use in unipolar depression and anxiety disorders. Additional reports concluded that lavender essential oil is neuroprotective in human and rat studies (Ayaz et al., 2017; López et al., 2017). Nevertheless, in research aimed at antimicrobial activity, lavender has also been shown to induce oxidative stress, as demonstrated by its capacity to modify membrane permeability in *Klebsiella pneumoniae* bacteria (Yang et al., 2020). Therefore, individuals with polymorphisms or impacted by epigenetic factors that reduce REST expression could evade the protective activities of lavender, and its use might even be detrimental to their cellular health.

&nbsp;We tested lavender in *C. elegans* *spr-4* mutants for two reasons. First, lavender is heavily enriched with terpenoids, which we hypothesized might stimulate stress-response pathways such as the WNT-β-catenin pathway, that in turn upregulates REST, as a target of this pathway (Nishihara et al., 2003). Second, we used mechanosensory assays involving response to soft touch to examine the effect of lavender on *spr-4* mutants because, according to the *C. elegans* neuronal gene expression compendium, CeNGEN (Taylor et al., 2021), *spr-4* is expressed in the ALM, AVM, and PLM neurons associated with controlling the escape response to gentle touch (Chalfie et al., 1985).

*C. elegans* strains with and without the *spr-4* mutation were exposed to extracts from fresh lavender plants at three concentrations and assayed for a response to gentle touch. &nbsp;Control worms, N2 wildtype (blue bars), did not display altered anterior or posterior touch response following exposure to any concentration of lavender extract (0.25, 1.0, and 2.6 mg/mL) ( Figure 1A, B). Likewise, *spr-4* mutant animals were not adversely impacted by lavender extract at low concentrations (0.25 or 1.0 mg/mL). However, at high concentrations (2.6 mg/mL), significant mechanosensory defects were uncovered for both anterior and posterior touch assays compared to N2 controls ( Figure 1A, B). These data are consistent with previously reported data where *spr-4 ( by105 )* nonsense mutation animals are more vulnerable to oxidative stress (Lu et al., 2014) and exposure to lavender in *Klebsiella pneumoniae* bacteria induce oxidative stress (Yang et al., 2020). Taken together, wildtype animals that express SPR-4 resist a lavender extract-associated vulnerability that is revealed by *spr-4* mutants at high concentrations.

## Methods

**Plant materials and lavender extraction** . Dry lavender ( *Lavandula angustifolia* ) flowers obtained from Starwest Botanicals was weighed to 2 g and thoroughly ground into a powder using a mortar and pestle. This powder was subsequently dispersed with an equal amount (2 g) of diatomaceous earth. Extractions were performed using an accelerated solvent extractor (Dionex ASE 150). The lavender/diatomaceous earth samples were packed into extraction cells, placed into the stainless-steel chambers, and extracted via an 8:2 methanol/H _2_ 0 solution at 120°C. The subsequent liquid extract was then transferred into a 500 mL round-bottom flask for rotary evaporation at 40°C, 120 rotations per minute to near-dryness. This residue was transferred into a 50 mL conical tube by resuspension with up to 2 mL of the 8:2 methanol/H _2_ O solution. The remaining solvent was then removed via air evaporation for approximately 2 hours, until only the residue remained at the bottom of the conical tube. This lyophilized extract was stored at 4°C. The weight was determined before resuspending it in 1 mL 0.05% DMSO. The lavender extract was then directly incorporated into cooled, liquid NGM media at 0.25, 1.0, or 2.6 mg/mL prior to pouring plates. The final concentration of DMSO was adjusted so that all plates received equivalent volumes of this solvent, including the 0 mg/mL control plates.

**Lavender exposures** . 60 mm NGM agar plates were prepared 48 hours before use. The lavender plates were seeded with *E. coli* OP50 after drying for 48 hours and then were dried for 30 minutes in a sterile hood with the lids cracked open before use. Three plates were prepared per strain and the experiment was repeated three times for each strain. A three-hour egg lay was performed onto the plates, and the resulting progeny were grown at 20°C for 3 days prior to the soft touch assay.

**Mechanosensation assay** . Assays were performed as previously described (Chalfie and Sulston, 1981; Chalfie et al., 1985). *C. elegans* sensitivity to soft touch was assayed by gently stroking the hermaphrodite animals on the posterior with an eyelash hair glued to the end of a Pasteur pipette.

Backward locomotion was induced by gently stroking the anterior of the animal (posterior to the nose, but not at the nose) with the eyelash followed by stroking the tail just below the anus to induce forward locomotion. A positive result for soft touch sensitivity was recorded if an animal ceased backward locomotion or began moving forward. This process was repeated 5 times per animal, and the number of positive responses to posterior soft touch out of 5 was recorded. A total of 30 worms were scored *per* biological replicate, with N = 3; n = 30 per extract tested and data represent the average of all three biological replicates with standard error of the mean (s.e.m.) calculated using GraphPad Prism, as previously reported (Griffin et al., 2019).

## Reagents

*Lavandula angustifolia* (from Starwest Botanicals, Sacramento, CA, USA)

methanol

diatomaceous earth

Dionex ASE 150 solvent extractor

Rotary evaporator

NGM agar plates

*C. elegans* strain N2 (Bristol)

*C. elegans* strain LA95 *spr-4 ( by105 )*

*E. coli* strain OP50 (saturated culture, previously grown in LB and stored at 4°C)
